# Effectiveness of a hydrogel dressing as an analgesic adjunct to first aid for the treatment of acute paediatric thermal burn injuries: study protocol for a randomised controlled trial

**DOI:** 10.1186/s13063-018-3057-x

**Published:** 2019-01-06

**Authors:** Maleea D. Holbert, Bronwyn R. Griffin, Steven M. McPhail, Robert S. Ware, Kelly Foster, Demi C. Bertoni, Roy M. Kimble

**Affiliations:** 1 Centre for Children’s Burns and Trauma Research, The University of Queensland, Brisbane, Australia; 2 Pegg Leditschke Paediatric Burns Centre, Lady Cilento Children’s Hospital, South Brisbane, Australia; 3 Centre for Functioning and Health Research, Metro South Health, Buranda, Australia; 4 School of Public Health & Social Work and Institute of Health and Biomedical Innovation, Queensland University of Technology, Brisbane, Australia; 5 Menzies Health Institute Queensland, Griffith University, Brisbane, Australia; 6 Paediatric Emergency Research Unit, Children’s Health Queensland, South Brisbane, Australia; 7 School of Nursing, Queensland University of Technology, Brisbane, Australia

**Keywords:** Paediatric, Burns, First aid, Hydrogel, Burnaid®, Re-epithelialisation, Analgesia, Dressing, Randomised controlled trial, Pain

## Abstract

**Background:**

Burns are a painful and traumatic experience, particularly in children. Reduced pain and anxiety positively influences re-epithelialisation rates in paediatric burn patients, however current literature fails to fully explain the effects of pain and anxiety and their links with wound healing. This study will determine if Burnaid® hydrogel dressing is an effective treatment for reducing pain in the acute period of a burn injury. It is hypothesised that a reduction in pain will then improve re-epithelialisation time in comparison to plastic wrap, which is standard practice at our institution — a metropolitan tertiary paediatric hospital located in Brisbane, Australia.

**Methods/design:**

A randomised controlled trial will be conducted to assess the effectiveness of Burnaid® as an analgesic adjunct to cold running water first aid for the treatment of paediatric burns. Participants will include children aged between 0 and 16 years with an acute thermal burn injury (total burn surface area < 20%) presenting to the Department of Emergency within 24 h of the burn occurring. Participants will be randomised into one of two groups: (1) Burnaid® hydrogel (intervention arm) or (2) plastic wrap (control arm). Participants will also be stratified into one of two groups based on factors that influence pain intensity: (1) high pain risk or (2) low pain risk. High pain risk factors include foot burns, hot coal/ash/fire pit burns, burn area greater than 5%, and circumferential burns. The primary outcome is the intervention’s effect on reducing acute pain. Secondary outcomes include days to re-epithelialisation, pulse rate, temperature, salivary cortisol and α-amylase, anxiety, and cost-effectiveness. Sample size calculations have shown that 36 participants will be recruited into each group.

**Discussion:**

This study will provide comprehensive data on the analgesic properties of Burnaid® as an adjunct to first aid for the treatment of acute paediatric thermal burns. If the intervention is effective in reducing pain, Burnaid® will be integrated as standard practice within the hospital’s Department of Emergency. This study replicates a real-world scenario in order to identify clinically significant analgesic and wound-healing effects.

**Trial registration:**

Australian New Zealand Clinical Trials Registry, ACTRN12617001274369. Prospectively registered on 5 Sept 2017.

**Electronic supplementary material:**

The online version of this article (10.1186/s13063-018-3057-x) contains supplementary material, which is available to authorized users.

## Background

Paediatric burn injuries pose a major epidemiological problem worldwide, with one-fourth of all burn injuries occurring in children under the age of 16, the majority of whom are under 5 years of age [1]. Comprehensive management of burn wounds includes a challenging spectrum of acute, chronic, traumatic and surgical wounds with a wide range of anatomical locations and depths. Improving acute paediatric burn management is critical because untreated pain is thought to contribute to long-term sensory issues such as chronic pain, paraesthesia, dysesthesia and psychological conditions [2]. Moreover, optimal pain management may have significant implications with respect to re-epithelialisation rates in children with burn injuries [3].

Historically, burn injuries have been viewed as one of the most severe forms of trauma, and burn pain is considered one of the most severe forms of acute pain [4, 5]. Burn treatment is also associated with significant pain and distress, and current standard treatments for such injuries have been insufficient in providing the analgesic needs for paediatric patients [6–9]. Paediatric burn patients undergo multiple painful and distressing procedures during their wound care and rehabilitation, which can cause severe long-term physiological and emotional effects [2]. If acute pain is not adequately managed, it can lead to altered long-term pain perception and maladaptive coping strategies, which can persist into adulthood [10–13]. Previous research also suggests that inadequate pain control can lead to the development of anticipatory anxiety for future medical procedures [9]. It is estimated that 10–20% of young patients who have sustained a burn injury go on to develop psychological disorders such as post-traumatic stress disorder [13, 14]. Therefore, optimising pain management during the acute phase of a paediatric burn is critical not only for the physical and cosmetic outcome of the injury but also for the child’s well-being at the time and in the long term.

### First aid

Correct first aid in the acute phase of a burn is essential for preventing further tissue damage, reducing pain in the acute phase and improving time to re-epithelialisation [15–20]. Immediate cooling via the use of cold running water (CRW) between 2 °C and 15 °C for 20 min (applied within 3 h of the initial injury) is the recommended gold standard first aid for thermal burns, according to the Australian and New Zealand Burn Association [15, 17, 21, 22]. After adequate CRW the burn must be covered to protect the wound, reduce pain and prevent hypothermia [8]. The use of a hydrogel dressing that aids in cooling and hydration to the burn wound could be an ideal adjunct to current first aid practice. Advanced wound-dressing technologies require clinical data to support their use, and currently there is limited empirical evidence to support the use of hydrogel dressings as an analgesic adjunct to first aid for the treatment of acute burn injuries. Moreover, there are no studies investigating the use of these dressings in the paediatric burn population.

### Use of hydrogels on burns

Current guidelines recommend burn wound covering after CRW first aid [8]. Acute burn wound coverings are required to be transparent and non-adherent and to have high application and removal ease. Plastic wrap fulfils these criteria and, excluding applications to the face, is a practical choice of dressing for acute burn injuries. In comparison to plastic wrap, Burnaid® hydrogel dressings (Mundicare, Sydney, Australia) may provide additional pain relief via an evaporative cooling effect and may thereby assist in wound healing [23–25]; however, empirical evidence is still limited. The Burnaid® hydrogel dressing comprises a 3-mm-thick, sterile, open foam polyester urethane pad that is impregnated with a propylene glycol gel which contains more than 90% purified water. Following the recognised standard of first aid treatment for acute burn injuries, Burnaid® is recognised as an adjunct to first aid whose aim is to reduce pain. Moist interactive wound dressings provide pain relief via an evaporative cooling effect and create a moist environment which soothes exposed nerve endings in the skin [26]. Hydrogel dressings provide a concomitant cooling and wound-covering function and dissipate heat from the burn wound, and dressings come in a wide range of sizes which can be applied to all areas of the body [23, 27, 28].

The use of hydrogel dressings as an acute treatment for burn injuries has increased in the pre-hospital setting over the past decade. Allison showed that 39% of emergency medical services (EMS) in the United Kingdom applied hydrogel dressings (e.g., Water-Jel, Water-Jel Technologies, Carlstadt, NJ, USA; Burnshield, Levtrade, Johannesburg, South Africa) during patient transport to hospital for burn injuries [29]. Walker and colleagues showed that 76% of UK fire and rescue services applied hydrogel dressings to cover burns, whereas 23% used plastic wrap as a burn wound dressing until EMS arrived [30]. Moreover, Cuttle and colleagues conducted an audit of first aid interventions for paediatric burn patients presenting to a tertiary burns centre located in Brisbane, Australia. This investigation found that Burnaid® hydrogel dressings were applied by ambulance services in 12.9% of cases [31]. In addition, authors of a 2013 retrospective investigation reported that 5% of paediatric burns patients who presented to a tertiary burns centre in Sydney, Australia, had Burnaid® hydrogel dressings applied as a first aid dressing [32]. A large retrospective investigation into pre-hospital paediatric burn care was conducted in 2014 using data from electronic ambulance response forms for patients aged between 0 and 5 years who were attended by the Queensland Ambulance Service from 2008 to 2010 [18]. Data were collected from 117 paediatric burn incidents occurring within the Brisbane, Townsville and Cairns regions. Burnaid® was the most commonly used dressing during patient transport and was applied to 55.6% of all paediatric burn patients. Brisbane paramedics applied Burnaid® dressings in 44.3% of paediatric burn cases, whereas ambulance services in North and Far North Queensland applied Burnaid® during patient transport in 72.7% and 60.9% of cases, respectively [18]. The difference in rates of use between regions further exemplifies the need for evidence-based guidelines for the use of hydrogel dressings in burn patients.

In a clinical investigation in which researchers evaluated the efficacy of hydrogel dressings in burn wound care, it was reported that hydrogel dressings conform well to the wound bed, are quick and easy to apply, and allow for wound inspection through the dressing [33]. In addition, it was observed that hydrogel dressings are effective for providing hydration to deep partial thickness burns and act to absorb exudate without becoming adherent to the wound bed. It was further reported that hydrogel sheet dressings are well tolerated by patients, who report no pain associated with dressing changes or between dressing changes [33]. The researchers in that study also commented on the paucity of evidence for the use of hydrogel dressings in burn care and their use in major burn wounds. This demonstrates the need for further investigation into the efficacy of hydrogel dressings for the acute treatment of burn injuries, so that evidence-based guidelines regarding their use and application can be developed. The potential that hydrogel dressings can reduce pain during the acute burn phase should not be underestimated in a clinical area where pain is most probably at its greatest. The primary aim of the present research is to assess the analgesic properties of Burnaid® hydrogel dressings as an adjunct to current first aid in comparison to plastic wrap, which is currently used to cover burn injuries following CRW.

### Effect of pain and inflammatory mediators on wound healing

The association between pain and wound healing is complex. In patients with significant tissue damage such as a burn, there is a prolonged and intense release of inflammatory mediators, and this can result in a state of hyperexcitability, which leads to an increased perception of pain [34]. It is evident that prolonged pain, such as that experienced by patients with significant burn injuries, can have maladaptive effects on wound healing time [35] and individual pain thresholds [2]. Prolonged release of inflammatory mediators from injured tissue acts to sensitise the inflamed area to pain via lowering the excitation threshold of nociceptors [34, 36]. This lowering of activation threshold (hyperalgesia) results in the perception of pain from typically non-painful stimuli and is often the case in burn patients who report increasing pain even where good progressive wound healing has occurred [36]. This further demonstrates the critical need to optimise pain management during the acute phase of a paediatric burn.

### Distraction, pain and wound healing

Several studies describe the relationship between decreased pain and improved re-epithelialisation rates in paediatric burn patients [3, 6, 37, 38]. A recent randomised controlled trial assessed the effectiveness of a procedural preparation and distraction device (Ditto™; Diversionary Therapy Technologies, Toowong, Australia) in reducing pain in paediatric burn patients during burn wound care [3]. This trial found that children who received the procedural preparation and distraction treatment reported significantly lower procedural pain scores (measured using the Wong-Baker Faces Pain Rating Scale) in comparison to patients who received standard care during their burn wound treatment (*p* ≤ 0.05). Parent and caregiver observational reports of procedural pain were also found to be lower in patients who received the preparation and distraction device (*p* < 0.001). Furthermore, children who received the Ditto™ device re-epithelialised an average of 2.1 days faster than children in the standard care group (CI, − 4.38, 0.10; *p* = 0.061). Although not statistically significant, this result was clinically significant because the 2-day improvement rate in re-epithelialisation in patients who received the Ditto™ treatment translated into a sizeable reduction in the need for scar monitoring: 26% of patients in the Ditto™ treatment group re-epithelialised beyond 14 days, in contrast to 48% of participants in the standard practice group.

### Objectives

The objective of the present study is to compare the effectiveness of plastic wrap (currently used to cover burns following CRW at Lady Cilento Children’s Hospital (LCCH; Brisbane) and Burnaid® hydrogel dressing to reduce pain intensity in children who have sustained a burn injury prior to the application of a standard burns dressing.

### Hypotheses

It is hypothesised that Burnaid® is more effective than plastic wrap at reducing pain intensity in the acute burn phase. It is also hypothesised that decreased pain experienced during the acute phase of the burn injury will speed up wound healing time and will result in a clinically significant reduction in days to full re-epithelialisation after application in children after a burn.

## Methods/design

### Design

**Fig. 1:**
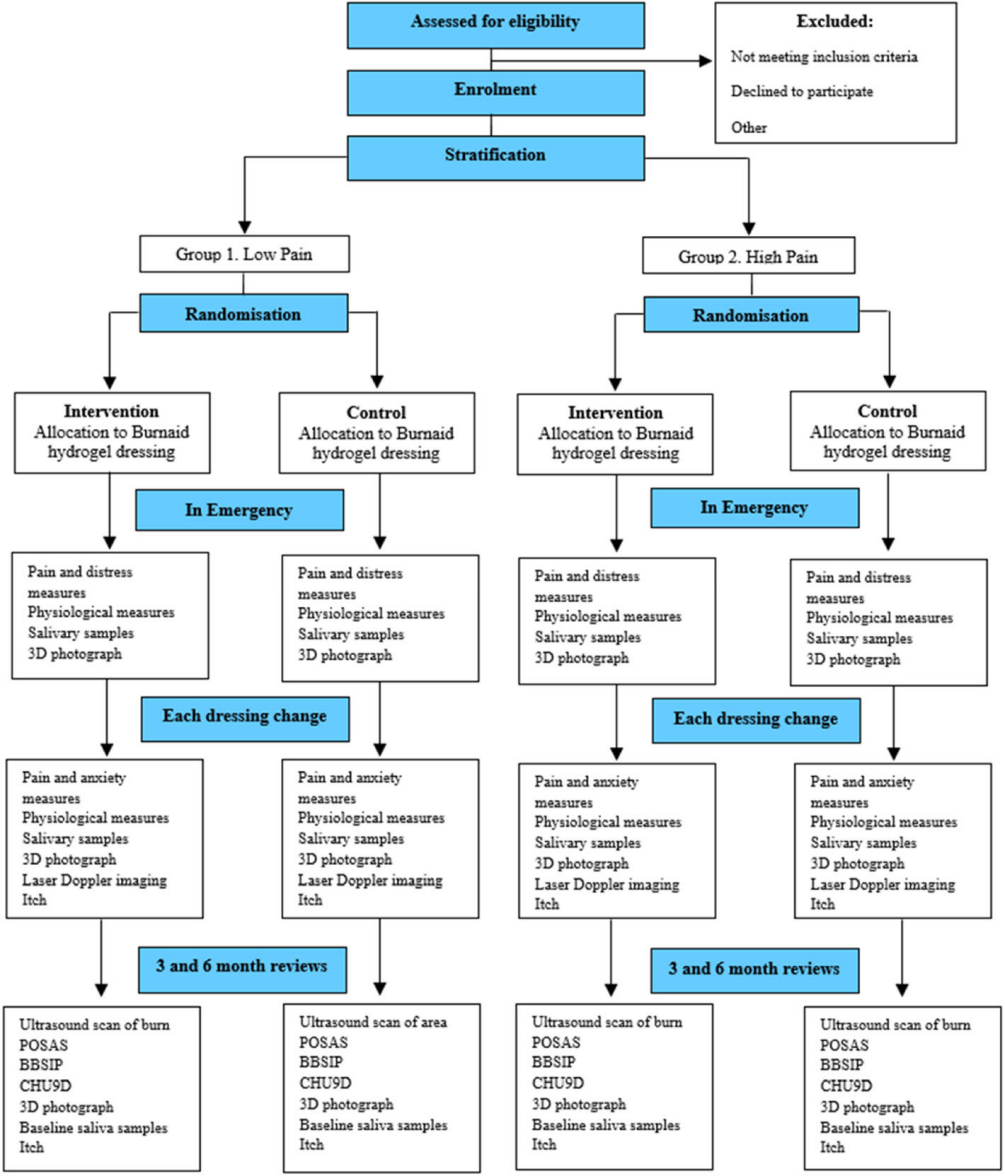
Data collection flow diagram

A single-centre, superiority, two-arm, parallel-group randomised controlled trial will be conducted to examine the effectiveness of Burnaid® hydrogel dressing as an analgesic adjunct to first aid in comparison to plastic wrap. The present study includes methods that have been modelled on successful prior trials conducted by members of this investigative team at the same participating facility as the present trial [39, 40]. The Consolidated Standards of Reporting Trials guidelines for randomised controlled trials will be followed, and the protocol adheres to the Standard Protocol Items: Recommendations for Interventional Trials guidelines (SPIRIT; *see* checklist in Additional file 1). Ethics approval for this project was obtained from LCCH and The University of Queensland human research ethics committees (HRECs). A flow diagram of the trial is provided in Fig. 1.

### Setting

Recruitment will be conducted in the Department of Emergency (DEM) and Pegg Leditschke Children’s Burns Centre Outpatient Department (OPD), located at LCCH, Brisbane. LCCH is categorised as a level 6 service, providing tertiary-level care for the state’s most critically and seriously injured children.

### Inclusion criteria

Children recruited into the study will be aged between 0 and 16 years and will have acquired an acute thermal burn injury that is < 20% of the total body surface area (TBSA). The child must present to the DEM or burns OPD within 24 h of sustaining the injury and must have no silver dressing application prior to this admission.

### Exclusion criteria

Exclusion criteria for mechanism of injury are chemical, electrical, inhalation or friction burns; children who do not arrive within 24 h of sustaining their acute burn injury; children who do not receive appropriate first aid treatment; children who received prior treatment of a silver dressing or ointment; children who are non-English-speaking; children with cognitive impairments; children who are ventilated or require initial wound care in theatre for debridement or grafting; current involvement with the Department of Communities (child safety); children with known sensitivity to hydrogels or hypersensitivity; and children who have co-morbidities that would potentially impair would healing (e.g., diabetes) or that may exacerbate itch and pain.

### Recruitment

Treating physicians/nursing staff of all children meeting the inclusion/exclusion criteria and presenting to the LCCH, Brisbane, will determine eligibility for enrolment in the study. Medical staff, once establishing the child’s eligibility, will then notify the parents/guardians and child of their eligibility to participant in the study. With parent/caregiver permission an investigator aligned with the study will discuss the trial with the parent/caregiver, seek informed consent from the parent/caregiver, and seek assent from the child (when appropriate). This trial will use a stratified sampling method taking into account factors which influence pain intensity in paediatric burn patients, based on anecdotal evidence and observations from paediatric burns surgeons and the unit’s experience (unpublished hospital data audit). Once informed consent is obtained participants will be stratified into one of two groups: (1) high pain risk or (2) low pain risk. Participants presenting to LCCH DEM or burns OPD with one or more of the following criteria will be placed into the high pain risk group:

- Foot burns (unilateral or bilateral)
- Hot coal/ash/fire pit burns
- Circumferential burns
- TBSA > 5%

All other participants who meet the inclusion criteria for the trial but do not present with one or more of the four aforementioned pain risks will be placed in the low pain risk group. Following stratification, children will be randomised to one of the two treatment groups. Randomisation will be undertaken using a computerised random number sequence-generating programme, and treatment allocation will be masked by the use of sealed, opaque, identical, serially numbered envelopes prepared by an independent party. The researchers will not be blinded to which group the child is in, because they will be collecting set measures throughout the acute burn period. At any point throughout the study period the parent/guardian or child can withdraw from the study without any implications for the child’s burn care, and all withdrawals and discontinuations will be recorded.

### Treatments

#### Intervention

Burnaid® hydrogel dressing will serve as the treatment intervention in this trial. Paediatric burn patients randomised to the intervention arm will have the hydrogel dressing applied following 20 min of CRW, and the dressing will remain on the burn for a minimum of 20 min. Burnaid® will be applied as per the manufacturer’s instructions, followed by standard burn care, typically consisting of wound cleaning, debridement and silver dressing application.

#### Control

Plastic wrap is the current acute wound covering applied to thermal burn injuries at LCCH, and it will serve as the control arm in this trial. Participants randomised to receive plastic wrap will have the dressing applied after first aid and will have the dressing remain on for a minimum of 20 min, followed by standard burn care.

### Data collection

**Fig. 2:**
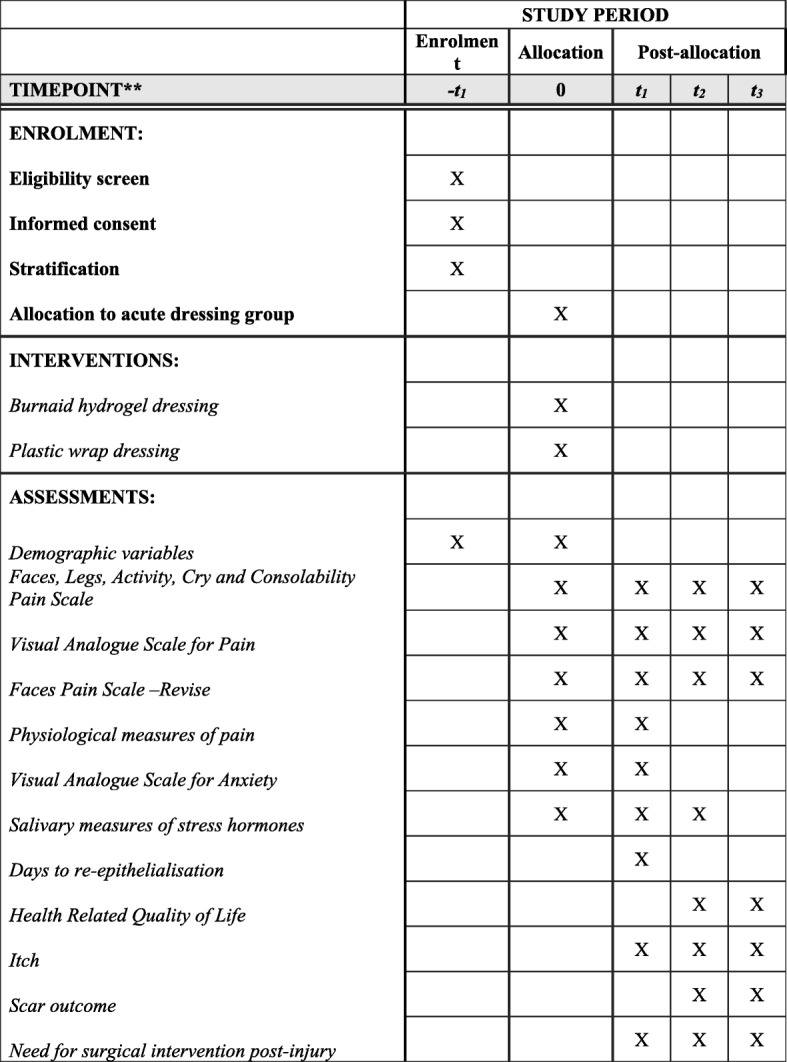
SPIRIT figure. SPIRIT schedule of study recruitment, intervention and assessments. Allocation to the intervention group will occur in the Department of Emergency at Lady Cilento Children’s Hospital upon patient admission following a thermal burn injury; t1 = 3–5 days after burn injury and each subsequent dressing change; t2 = 3 months after burn injury; t3 = 6 months after burn injury

Information regarding patient demographics and clinical details will be obtained from the parent/guardian, child (where age appropriate), and patient chart to record mechanism of injury, affected area, burn TBSA, what first aid was provided, form of transport, and time to presentation (*see* SPIRIT figure in Fig. 2). Apart from which type of dressing the child is receiving prior to the application of silver dressings, all participants will receive the standard medical care as would those not in the trial.

Following patient enrolment and randomisation, acute measures will be collected in LCCH DEM. Acute measures will include pain (measured using the Faces Pain Scale–Revised [FPS-R]; Face, Legs, Activity, Cry, Consolability scale [FLACC]; and visual analogue scale [VAS]), pulse rate, temperature, respiratory rate, and salivary cortisol and α-amylase, which will be recorded by the primary investigator. Acute measures will be collected before and after the application of the randomised dressing and before and after silver dressing application from the child, parent/guardian, and nursing staff. In addition, peak FLACC scores during wound cleaning and debridement will be documented, as will all analgesia and distraction techniques. All participants will have their burn wounds photographed using 3D imaging after wound debridement in the DEM to assess burn area. Patients (where appropriate) and parents/caregivers will be sent home with a diary to record pain scores and medications required prior to their first change of dressing (COD), which will typically occur 3 days after injury. Participants and parents/caregivers will be asked to bring this home diary with them to their first dressing change in the burns OPD.

Data collection in the burns OPD will continue through to the child’s treating clinician classifying the burn wound as 95% re-epithelialised. Measures collected in the burns OPD at each dressing change appointment will include pain (measured using FPS-R, FLACC, and VAS), pulse rate, temperature, respiratory rate, and salivary cortisol and α-amylase at each dressing change appointment. These measures will be collected before and after silver dressing removal and before and after the application of new silver dressings (*see* Fig. 4). Anxiety and itch will also be assessed at each COD appointment. At the first COD appointment, all participants will have their burn wound scanned using a non-invasive treatment referred to as laser Doppler imaging to measure burn depth and vascularity of the affected area. In addition, all participants will have a 3D image of their burn taken at each COD to assess the area of the wound and its rate of re-epithelialisation.

Following wound re-epithelialisation and discharge from the burns OPD, scar outcome will be assessed at 3 and 6 months post-burn for all participants. At each 3- and 6-month follow-up, patients will have their burn wounds scanned using ultrasound to assess scar thickness; 3D imaging to assess scar area; DSM ColorMeter (Cortex Technology, Hadsund, Denmark) to examine scar colour and pigmentation; and Patient and Observer Scar Assessment Scale (POSAS), Brisbane Burn Scar Impact Profile (BBSIP) and Child Health Utility 9D Index (CHU9D) to measure health-related quality of life. Thy will also undergo itch assessment, and saliva samples will be collected to examine baseline cortisol and α-amylase levels.

### Primary outcome measure

**Fig. 3:**
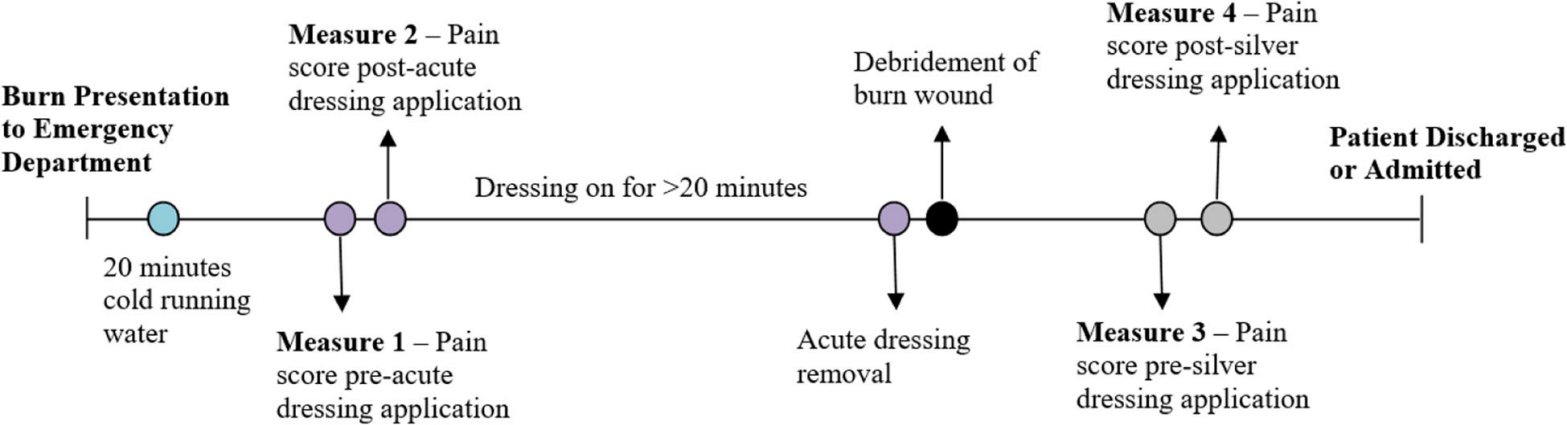
Acute pain assessment in the Department of Emergency. Following cold running water first aid, pain will be assessed before and after the application of the randomised dressing, peak pain scores during wound cleaning and debridement, and before and after silver dressing application. Pain scores will be collected from the patient (where appropriate), observational pain scores will be collected from parents and caregivers, and Face, Legs, Activity, Cry, Consolability scale scores will be collected from nursing staff

The primary outcome of this trial is pain intensity after acute dressing application (*see* Measure 2 in Fig. 3). Observational reports from nursing staff using the FLACC scale will be used to measure this primary study outcome for all participants enrolled in this trial.

#### Ancillary pain measures

Pain intensity will be assessed before and after hydrogel dressing application and before and after silver dressing application, using self-report scales, parental/carer-specific, and nursing-specific pain scales (*see* Fig. 3). The set points of data collection will be recorded on a data collection form for patient and external observers to communicate pain intensity in a more accurate manner. The three physiological and psychological age-specific scales outlined below have been selected to allow assessment of pain and distress.

1. The FPS–R will be used to assess pain in children aged 4 years and older. The FPS–R is a self-rated, self-administered scale that uses images of facial expressions to assess pain intensity in children [41, 42]. The FPS–R has been shown to be superior to other faces scales [43] and has been validated in the literature as having a strong positive correlation (*r* = 0.93) with the visual analogue scale for pain (VAS-P) in children aged between 5 and 12 years [44]. Authors of a systematic review of self-report pain intensity measures for use in paediatric clinical trials reported that the FPS–R appears to be the most psychometrically sound pain assessment tool for children aged between 4 and 12 years [42]. Furthermore, a 2010 systematic review of the FPS–R for child self-reporting of pain intensity concluded that the FPS–R is recommended as a research tool because of the measure’s utility and psychometric features [45].
2. The FLACC scale [46] will be used by nursing staff to assess pain and distress levels. The FLACC is an observational behavioural scale that has been validated for pain assessment in children aged between 2 months – 7 years [46]. Observational pain scales such as the FLACC are widely used in paediatric clinical research [47]. A prospective observational investigation conducted in 2012 assessed the use of the FLACC scale to measure pain and distress in patients aged between 6 and 42 months (*N* = 125). This study found that FLACC scores can be high during non-painful procedures, as well as during the restraining phase of procedures [47]. These findings further support the notion that the FLACC assesses a composite of pain and distress in young children.
3. VAS-P for children aged 8 years and older and visual analogue scale observer (VASobs) for parent/guardian reports will be used. These scales have been chosen because they are age-specific and reliable and have been validated in recent literature. VASs have been validated and frequently used to measure pain in adult populations and in paediatric populations as young as 5 years of age [48–50]. When quantifying pain intensity in pre-verbal and early-verbal children, who are unable to self-report, the VASobs repeatedly appears in the literature as a proxy for self-reports of pain [50–52]. Earlier studies have demonstrated that young burn patients can readily grasp the concept of quantifying their pain using anchored scales and even in moments of extreme discomfort are able to continue giving pain scores [53].

**Fig. 4:**
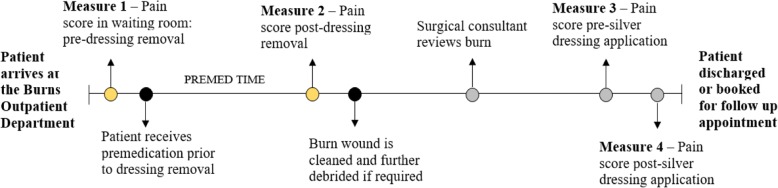
Pain assessment during change of dressing follow-up appointments. All participants will have their pain assessed in the waiting room of the Pegg Leditschke Children’s Burns Centre Outpatient Department prior to premedication for their follow-up dressing changes. Pain scores will also be measured subsequent to silver dressing removal, prior to the application of new silver dressings, and after application of the new silver dressing

Physical measures of pain and distress will also be recorded at each of the measurement points depicted in Figs. 3 and 4. These measures include pulse rate, respiratory rate and temperature because increases in these physiological measures have been shown to be indicative of pain and distress.

### Secondary outcome measures

#### Days to re-epithelialisation

The number of days from the date of the initial burn until 95% wound re-epithelialisation occurs, the surface area of the affected area, and the percentage of wound re-epithelialisation will be calculated using three methods: (1) clinical judgment of the treating consultant, (2) 3D photography (3D LifeViz™ System; QuantifiCare, Valbonne, France) and analysis using specialist computer software, and (3) blinded review of the 3D photographs by a panel of burn specialists. The 3D LifeViz™ System will be used to photograph all burns upon first presentation to the hospital following wound debridement and at each subsequent burns OPD visit for dressing changes and wound care. This will be performed for all participants until burn wounds are deemed to be 95% re-epithelialised or are grafted. Research has found 3D photography to be a valid and reliable measure of acute burn wound surface area in an Australian paediatric population [54, 55]. In order to ensure accurate measurement calibration in the associated DermaPix™ software package (QuantifiCare), a ruler will also be included in all 3D photographs. The 3D images will be analysed by the primary investigator using the DermaPix software programme to determine the surface area of the burn and the area of re-epithelialisation of the wound at each COD. A panel of burn specialists, which will be composed of consultants and nursing staff, will perform a blinded review of the 3D images and will assess the level of re-epithelialisation and appearance of each participant’s burn, mapping out the edges of the wound and all unhealed areas. We will further examine concordance between the burn specialist’s clinical assessment and the DermaPix mapping programme regarding re-epithelialisation.

#### Staff and caregiver perspectives on dressings

Perception of ease of dressing application, removal, flexibility, and conformity will be rated by medical and nursing staff using a self-reported 0–10 Numeric Rating Scale (NRS) for both the Burnaid® hydrogel dressing and plastic wrap groups. Clinical staff will also be surveyed again using the same NRS after recruitment has ceased in order to examine any changes in their perception of either acute wound covering. In addition, parent/caregiver and patient (when appropriate) perception of comfort, ease of movement, and ease of dressing application/removal will be measured using an NRS. It is noted that ease of dressing measurements in the DEM will be confounded owing to lack of blinding and as a result of the variable nature, size, and anatomical location of the areas to be dressed.

#### Scar/skin condition at 3 and 6 months

At 3 and 6 months after the date of injury, a face-to-face follow-up will be performed with all participants (intervention and standard care groups) to assess the skin and scar characteristics of the affected area. This assessment will involve (1) 3D camera imaging (3D LifeViz™ System), which will be used to determine scar area and volume above the skin; (2) ultrasound scanning (BT12 Venue 40 MSK; GE Healthcare, Chicago, IL, USA), which will be used to measure the height of the scar; and (3) the POSAS measurement tool, which will be used to assess scar severity. The POSAS will be completed with the patient (if aged 6 years or older) and the parent/caregiver, and it will document observer scale items of scar thickness, vascularity, pliability, pigmentation and relief in addition to patient scale items of itch, pain, colour, stiffness, thickness and irregularity. In addition, DSM II Skin ColorMeter analysis will be performed to assess the lightness, redness or erythema, and pigmentation of the scar. The DSM II ColorMeter is an objective tool that uses tri-stimulus reflectance colourimetry and narrow-band photometry to assess scar characteristics, and it has been demonstrated to have inter-observer reliability intra-class correlation coefficients of 0.94 for lightness, 0.94 for erythema and 0.83 for pigmentation in scar assessment [56].

#### Pain frequency

The BBSIP will be used to assess the intensity and frequency of sensations, including pain, tightness and discomfort, in addition to health-related quality of life attributes which are specific to individuals with burn scars. The BBSIP provides unique insight into health-related quality of life in children with burn scarring, in addition to the concerns of parents/caregivers of children with burn scaring. It has been shown to have preliminary content validity, and further research is currently being conducted in children, adults and parents/caregivers of children with burn scars [57].

#### Itch

The Toronto Paediatric Itch Scale (TPIS) will be used to assess pruritus in patients younger than 5 years of age. The TPIS is a behaviour-anchored post-burn itch scale which has been demonstrated in the literature to be a valid and reliable measurement tool with a moderate inter-observer agreement (Cohen’s kappa of 0.52, *p <* 0.001) [58]. Parents/caregivers of patients younger than 5 years old will complete the TPIS, which rates itch behaviours on a scale of 0 (indicating absence of itch) to 3 (which represents severe itch with significant disruption). In addition to the aforementioned factors, this scale was selected because it has been shown to be user-friendly, intuitive and reliable for use in infants and children as young as 5 years old or less [58]. Participants 5 years of age and older will self-report their itch intensity using a 0–10 NRS. In addition, all caregivers will complete the itch component of the BBSIP. Itch will be assessed at the first COD and each follow-up until the wound is deemed re-epithelised, and it will also be assessed at 3- and 6-month reviews.

#### Health-related quality of life

The CHU9D is a generic preference-based instrument designed for use in children and adolescents to assess how their health affects their life. The CHU9D will be used to measure health-related quality of life in a way that is suitable for use in cost-utility analyses. Dimensions of the CHU9D include worry, sadness, pain, fatigue, annoyance, schoolwork/homework, sleep, daily routine and ability to partake in activities. Preference weights applied to these nine attributes enable the calculation of a multi-attribute utility score where 0 represents death and 1 represents full health [59]. The CHU9D has been developed and validated for young people aged between 7 and 17 years, and it has been shown to have good construct validity within an Australian adolescent population [60]. At each 3- and 6-month post-burn follow-up, children will self-complete the CHU9D as appropriate, and a parent/caregiver proxy-report will be completed for all participants (children of all ages).

#### Stress and anxiety

Stress is a secondary outcome and will be quantified via salivary cortisol and α-amylase. Salivary cortisol is often used as a biomarker to assess stress response and is considered to be a reliable index of hypothalamic-pituitary-adrenal axis activity [61]. The measurement of cortisol levels in saliva is the preferred method of assessment for researchers, particularly in a paediatric population; salivary analysis is non-invasive and pain-free in comparison to blood sampling and therefore does not induce additional stress and trauma (and potentially higher cortisol) as occurs with serum sampling [62]. Moreover, there is a strong correlation between cortisol levels in saliva and unbound cortisol in plasma [63]. Salivary α-amylase is also said to be a reliable, valid and sensitive biomarker for stress-related changes in the autonomic nervous system and is a proxy for noradrenaline, which is a sign of sympathetic adrenomedullary system activation [64].

Participants will place a SalivaBio Oral Swab™ (Salimetrics Europe Ltd., Newmarket, UK) under their tongue for 2 min for saliva collection. Salivary stress biomarkers will be collected at the following time points:

1. At first presentation to the DEM or burns OPD subsequent to the application of either Burnaid® hydrogel dressings or plastic wrap
2. Immediately after the removal of the Burnaid® or plastic wrap dressing
3. Before application of silver dressings
4. Immediately after the application of silver dressings
5. Immediately before premedication prior to the patient’s first COD
6. Immediately before and after silver dressing removal
7. After application of the new silver dressings
8. Three months after burn injury to obtain a baseline

Parents/caregivers and patients (where appropriate) will also complete a saliva collection survey which documents variables pertinent to salivary analysis. Such variables include all medications given to the patient, time the patient last woke up, time the patient last brushed their teeth, all food/drink/gum consumed by the patient during the previous hour, time the participant last consumed caffeine, pertinent smoking/tobacco history, and sample collection time. The date, time and volume of saliva collection will be recorded in the laboratory, and samples will be refrigerated at 4 °C and processed within 3 days of collection. Samples will be spun in a centrifuge at 1400 × *g* at room temperature for 15 min, and the saliva will be frozen at − 80 °C until analysis. Salivary cortisol and α-amylase will be quantified using enzyme-linked immunosorbent assay kits (Stratech Scientific, Ely, UK), with saliva samples analysed in triplicate to ensure accurate results.

#### Healthcare resources and costs

Healthcare resource use will be collected from the perspective of a health service provider and costed at market rates using methods that have previously been employed in trials by members of this research team at this participating centre [65, 66]. This includes recording trial intervention-related resource use (e.g., the types and amounts of acute wound dressings used) as well as other burn-related healthcare resources that may be important to a health service when deciding which of the interventions to implement in clinical services providing care for children with acute burns. Clinician labour time will be recorded for each participant and costed at state award rates for each respective discipline.

#### Blinding

Owing to the nature of the trial, blinding will not be possible, because treating clinicians, nursing staff, patients and their families will be aware of the intervention. The primary investigator will be present when the acute wound dressings are being applied and removed to obtain pain scores and additional measures from the participant, caregiver and nursing staff. The acute wound covering applied to each participant cannot be masked within this environment, and therefore investigator blinding will not be possible. In order to incorporate an element of blinding in the trial, an expert panel of burn wound specialist will conduct a blinded review of 3D photographs for all participants to assess burn wound depth and re-epithelialisation at each dressing change until complete wound healing. Wherever possible during data collection analysis, treatment groups will be de-identified.

#### Adverse effects

The proposed intervention is considered to be part of standard pre-hospital care and is currently used by Queensland Ambulance Services, despite Burnaid® products not being used at the LCCH DEM and burns OPD. Therefore, minimal adverse events are expected. Known potential adverse events (such as hypothermia, infection, haematoma, excessive exudates, unpleasant sensations, allergic reactions, chondritis, and disruption of the skin on dressing removal) have a standardised management protocol at LCCH DEM and burns OPD. In addition, children with known adverse reactions to hydrogels will be excluded from the trial. Adverse effects of the hydrogel dressing will be monitored by reviewing patient medical records and via self-report data from parents/caregivers, participants (where appropriate), and treating clinicians. All adverse events will be reported to the clinical health service and the overseeing HREC. If a consultant believes that Burnaid® hydrogel dressings were not appropriate for a patient’s treatment, discontinuation or alteration of treatment will be at their discretion, and data collection for such participants will cease from that time point.

### Data monitoring

Regular team meetings will be held to monitor the progress of the study and set timelines and will allow for any issues to be discussed and resolved. All data will remain secure and de-identified, which the primary investigator will ensure in addition to data cleansing and circulation of results/outcomes.

### Data storage

Collected electronic data will remain secure through password protection on University of Queensland servers. Filing cabinets containing patient data will remain locked and secured within the swipe card-accessed Centre for Children’s Health Research. All data collected for the purpose of this investigation will be accessible only by the primary investigator and approved names on the ethics application form. Data collected and used for the trial will be de-identified, cleaned and checked. Any missing data will be coded as missing, unknown or not applicable before being locked for analysis. Data will remain secure for 15 years as per the requirements of The University of Queensland HREC.

### Sample size

The sample size estimate was calculated on the basis of the primary outcome of pain intensity after dressing application. Previous researchers assessing paediatric burn pain reported that pain scores within each subject group were normally distributed with an SD of 2.4 [37]. To detect a significant between-group difference of 1.8 in pain scores after dressing application, 29 experimental participants and 29 control participants will need to be recruited in order to reject the null hypothesis that the population means of the experimental and control groups are equal with probability (power) of 0.8. The type I error probability associated with the test of this null hypothesis is 0.05. With up to a potential 20% loss to follow-up, the calculated target sample size is equal to 72 participants. LCCH treats approximately 25–34 children per month in the DEM for acute burn injury. On the basis of these estimates and a historical 70% success rate for trial recruitment within LCCH, it is predicted that around 17 patients will be recruited per month. Therefore, it is estimated that data collection should be completed within 12 months from recruitment commencement.

### Data analysis

The data set will be analysed using IBM SPSS Statistics software (version 22; IBM, Armonk, NY, USA) and Stata software (StataCorp, College Station, TX, USA). Descriptive statistics will be calculated for all outcomes. Univariate parametric analyses (e.g., *t* test, chi-square test) or alternative non-parametric equivalent tests (e.g., Mann-Whitney *U* test) will be used to assess differences between groups at baseline where appropriate. Between-group differences in outcomes will be analysed using regression models. When analyses include multiple measures on the same participant, mixed effects methods will be used to account for probable non-independence in observations. When outcomes are on an interval scale, linear models will be used. When outcomes are binary, logistic models will be used, and when outcomes are recorded as count data, Poisson models will be used. Sensitivity analyses will be undertaken using multiple imputation methods, where it can be assumed data are missing at random. Analysis of differences between groups, and also differences within groups over time, will be undertaken. Analyses will be done on an intention-to-treat basis. Significance will be set at *p* < 0.05.

## Discussion

Pain and distress following first aid remains a major challenge when treating acute paediatric burn injuries, and literature shows that increased pain and stress can have maladaptive effects on wound healing [35, 37, 67] and patient outcomes [68]. Although Burnaid® hydrogel dressings are frequently being used by Queensland Ambulance Services during patient transport to hospital for burn injuries [18], a limited number of high-level studies have been conducted examining the clinical utility of these dressings, particularly in relation to analgesic properties and wound-healing effects. The widespread use of hydrogel burn dressings (such as Burnaid®, BurnShield®, and Water-Jel®) is alarming owing to the lack of research supporting their use as a first aid dressing [69]. This randomised controlled trial will provide high-level evidence to determine if Burnaid® hydrogel dressing provides superior pain relief in comparison to plastic wrap as an acute burn wound covering applied after first aid in paediatric patients with thermal burn injuries. Establishing which dressing is more effective in reducing acute pain is critical to clinical care and also to facilitate further evidence-based guidelines in this field.

Burns are a painful and traumatic experience, particularly in children. Considering the adverse psychological and physiological effects secondary to pain, optimal pain management should be viewed as an essential and requisite component in acute paediatric burn treatment [9]. Reduced pain and anxiety positively influences re-epithelialisation rates [3], and time to re-epithelialisation is a good predictor of scar outcome in patients with burn injuries [70]. This further validates attempts to speed up re-epithelialisation rates. Even if hydrogel products are more expensive than current standard acute wound coverings, the cost of treating adverse scarring is substantial in resources and time, as well as in cosmetic outcome for the patient. Burnaid® hydrogel dressings have been shown to be effective evaporative cooling agents, which is why these dressings are hypothesised to have an analgesic effect during acute burn care [69].

## Trial status

Recruitment will commence at the beginning of September 2017, and it is expected that recruitment will take approximately 12 months to complete, with final data collection occurring in January 2019.

## Additional file

Additional file 1: SPIRIT checklist. (DOC 124 kb)

## Abbreviations

BBSIP: Brisbane Burn Scar Impact Profile
CHU9D: Child Health Utility 9D Index
COD: Change of dressing
CRW: Cold running water
DEM: Department of Emergency
EMS: Emergency medical services
FLACC: Face, Legs, Activity, Cry, Consolability scale
FPS-R: Faces Pain Scale–Revised
HREC: Human Research Ethics Committee
LCCH: Lady Cilento Children’s Hospital
NRS: Numeric Rating Scale
OPD: Outpatient department
POSAS: Patient and Observer Scar Assessment Scale
SPIRIT: Standard Protocol Items: Recommendations for Interventional Trials
TBSA: Total body surface area
TPIS: Toronto Paediatric Itch Scale
VAS: Visual analogue scale
VASobs: Visual analogue scale observer
VAS-P: Visual analogue scale for pain
